# Anthelmintic Potential of Thymoquinone and Curcumin on *Fasciola gigantica*

**DOI:** 10.1371/journal.pone.0171267

**Published:** 2017-02-02

**Authors:** Rizwan Ullah, Abdur Rehman, Mohd Faraz Zafeer, Lubna Rehman, Yasir A. Khan, M. A. Hannan Khan, Shahper N. Khan, Asad U. Khan, S. M. A. Abidi

**Affiliations:** 1 Section of Parasitology, Department of Zoology, Aligarh Muslim University, Aligarh, India; 2 Interdisciplinary Brain Research Centre (IBRC), J. N. Medical College, Aligarh Muslim University, Aligarh, India; 3 Interdisciplinary Biotechnology Unit, Aligarh Muslim University, Aligarh, India; Wayne State University, UNITED STATES

## Abstract

Fasciolosis an economically important global disease of ruminants in the temperate and tropical regions, caused by *Fasciola hepatica* and *F*. *gigantica*, respectively, also poses a potential zoonotic threat. In India alone it causes huge losses to stakeholders. Anthelmintics including triclabendazole have been used to control this menace but the emerging resistance against the available compounds necessitates identification of novel and alternative therapeutic measures involving plant derived natural compounds for their anthelmintic potential. Thymoquinone (T) and curcumin (C), the active ingredients of *Nigella sativa* and *Curcuma longa* respectively have been used as antiparasitic agents but the information on their flukicidal effect is very limited. Adult flukes of *F*. *gigantica* were *in vitro* exposed to different concentrations of thymoquinone and curcumin separately for 3h at 37+ 1^°^C. A significant (p<0.05) reduction in the worm motility at 60 μM concentration of both T and C was observed though all the worms remained alive after 3h exposure, whereas the effect on egg shedding was statistically insignificant. Pronounced tegumental disruptions and erosion of spines in the posterior region and around the acetabulum was evident. A significant (p<0.05) decrease in glutathione-S-transferase and superoxide dismutase activity and reduced glutathione (GSH) level was observed, while protein carbonylation increased differentially. A significant inhibition of CathepsinL (CatL) gene expression in thymoquinone treated worms was also evident. Further, *in silico* molecular docking of T and C with CatL revealed a stronger interaction of curcumin with the involvement of higher number of amino acids as compared to thymoquinone that could be more effective in inhibiting the antioxidant enzymes of *F*. *gigantica*. It is concluded that both the compounds understudy will decrease the detoxification ability of *F*. *gigantica*, while inhibition of CatL will significantly affect their virulence potential. Thus, both thymoquinone and curcumin appeared to be promising anthelmintic compounds for further investigations.

## Introduction

The liver fluke species, *Fasciola hepatica* and *Fasciola gigantica*, responsible for liver rot disease, fasciolosis, are widespread among ruminants in Asia, Africa and parts of Europe [1]. The disease incurs huge economic losses worldwide [2] including India where animal wealth contributes enormously to the agrarian based economy. Though fasciolosis is primarily of veterinary importance but human infections have also been reported throughout the world and a huge population is at risk [3,4]. In India where the tropical species, *F*. *gigantica*, is more common, the human liver fluke infections have also been reported from different regions [5–10]. Both adult and juvenile flukes of this species can be effectively treated by the flukicide, triclabendazole (TCBZ), but there is an emerging trend of anthelmintic resistance in liver flukes [11]. Numerous cases of anthelmintic resistance in liver flukes have been documented from across the world [12] therefore, there is an emergent need to identify novel and more effective alternatives along with validation of new drug and vaccine targets [4,13,14] such as cysteine proteases including cathepsins and glutathione-S-transferases which have been validated through functional genomics and proteomic approaches [14,15]. Both cathepsins and GSTs play vital role in the virulence and survival of the flukes. Cathepsins are predominantly involved in host tissue penetration, evasion of host immune responses and establishment within the host while GST’s are mainly involved in the detoxification process by helping the conjugation of glutathione with xenobiotic compounds, therefore, these enzymes have been considered as important drug targets as well as vaccine candidates [16–18].

An increased resistance to conventional drugs have been considered as the major drivers of drug discovery [19] and an increased understanding of phytotherapeutics together with the availability of the structural and functional information of the parasite molecules and the plant products further drive the search for a novel anti-parasitic agent. A number of plant derived natural products including the natural oils and their active ingredients have been shown to possess anti parasitic activity [20–26]. The dietary ingredients *Curcuma longa* (turmeric) and *Nigella sativa* seeds (Kalonji), commonly consumed as spices in the Asian sub-continent are the source of active ingredients, curcumin and thymoquinone respectively. Both these compounds posses enormous therapeutic potential as evident from their use to treat a wide variety of ailments [21,23,26] and they exhibit anti-parasitic activity as well under both *in vivo* and *in vitro* conditions [21,23,25,27–31]. However, most of these studies on parasites are restricted to a few protozoan species like *Plasmodium*, *Trypanosomes* and *Leishmania* [20,24,25] or helminthic species such as *Schistosoma mansoni*, filarial worm *Setaria cervi* and some cestodes [21,23,28–30]. Though anthelmintic activity of crude *Nigella sativa* oil and curcumin has been determined against various parasites, [23,32] but the efficacy of active components, thymoquinone and curcumin, has not been established against the tropical liver fluke, *Fasciola gigantica*. Since both these compounds target multiple signalling molecules and metabolic pathways [23,31] therefore, in the present study worm motility, egg shedding, ultrastructural changes in the tegument, activity of key enzymes like glutathione-S-transferase, superoxide dismutase, level of reduced glutathione, cysteine protease gene expression have been investigated following treatment of adult *F*. *gigantica* worms with curcumin and thymoquinone. Further, molecular interaction of the two compounds understudy with CathepsinL enzyme has also been investigated *in silico* in order to understand their efficacy.

## Materials and Methods

### Collection of worms and their treatment with thymoquinone and curcumin

The adult flukes of *Fasciola gigantica* were obtained from infected livers of buffaloes freshly slaughtered at the local abattoir maintained by the municipal corporation, Aligarh and thoroughly rinsed in Hanks’ saline (HBSS) pH 7.4 before separate incubations in RPMI 1640 medium containing different concentrations of thymoquinone and curcumin. The controls were incubated without the test compounds. A total of 5 worms were incubated in 25 ml of the medium with or without test compounds for 3 hours at 37°C. Thereafter worms were rinsed in fresh RPMI 1640 and then a 10 per cent homogenate (w/v) was prepared in 100 mM Phosphate buffer, pH 7.4, using glass tissue homogenizer, followed by centrifugation at 20,000xg in a refrigerated centrifuge (Eppendorf, Germany) for 15min at 4°C. A clear supernatant was collected for various assays or stored at -80°C for subsequent use. Both thymoquinone and curcumin were purchased from Sigma-Aldrich (St. Louis, USA).

### Parasite motility assay

After the incubation of worms in different concentrations of the T and C, worm motility was recorded at every half an hour interval until 3 hours post incubation using the motility scale of 0–5 as described by Stepek et al. [33] where 0 means complete loss of motility; 1 denotes movement in worms only when prodded; 2 denotes worms active only at ends; 3 means less movement throughout the body; 4 depicting active worms and 5 being highly motile worms. The motility of control worms was recorded in the absence of the test compounds.

### Egg shedding

Following incubation of worms in the test compounds (T and C), egg shedding experiments were conducted by incubating the worms in RPMI 1640 medium. Eggs were collected from the incubate and counted to check for any effect on the egg laying efficiency of the treated worms as compared to the untreated control worms.

### Tegumental surface changes

The ultrastructural changes in the tegumental topography of worms treated with the test compounds (T and C) were investigated by scanning electron microscopy (SEM) according to the procedure as described by Shareef et al. [34]. Briefly, after incubation the treated and untreated worms were rinsed in fresh RPMI 1640 medium and then flat fixed for 30 minutes followed by fixation of the worms in the freshly prepared 4% (w/v) glutaraldehyde, buffered with 0.1 M sodium cacodylate (pH 7.4) containing 3% (w/v) sucrose for 4 h at 4°C. The parasites were then washed 4 times with sodium cacodylate buffer (pH 7.4). The flukes were dehydrated in ascending grades of ethanol, dried in hexamethyldisilazane, mounted on stubs, sputter coated with gold and viewed on JSM 6510LV, JEOL scanning electron microscope operating at 15 keV at USIF facility, A.M.U., Aligarh.

### Protein estimation

Protein content of the control and drug treated worms was estimated by the dye binding method [35] using bovine serum albumin as standard.

### Assay of glutathione-S-transferase (GST)

GST assay was carried out according to the method of Habig et al. [36] using 10 mM GSH and 1 mM CDNB (1-Chloro-2,4-dinitrobenzene) as substrate. Assay was initiated by adding 50 μl of protein sample in 0.1 M Phosphate buffer (pH 7.4). The enzyme activity was calculated as nmoles of CDNB conjugate formed/min/mg protein using a molar extinction coefficient of 9.6x10^3^ M^-1^cm^-1^.

### Reduced glutathione assay

Reduced glutathione levels were estimated as total acid soluble sulfhydryl concentrations colorimetrically using Ellman’s reagent [5, 5’-dithiobis-(2-nitrobenzoic acid) or DTNB] according to the procedure modified by Jollow et al. [37]. Proteins were precipitated with sulphosalicylic acid (4%) in the ratio of 1:1. The samples were kept at 4°C for 1 hour and then subjected to centrifugation at 1,700 g for 15 min at 4°C. The assay mixture contained 0.4 ml supernatant, 2.2 ml of 0.1 M sodium phosphate buffer (pH 7.4) and 0.4 ml of DTNB, making a total volume of 3 ml. The optical density of reaction product was read immediately at 412 nm on a spectrophotometer (Taurus Scientific) and the results were expressed as μmoles of GSH/gram tissue.

### Analysis of superoxide dismutase (SOD) activity

SOD activity was measured according to the method of Marklund and Marklund [38] using a reaction mixture of 3 ml having 2.85 mL of 50 mM Tris-cacodylate buffer (pH 8.5) and 50 μl of sample. Assay was initiated by addition of 100 μl of 0.13 mM pyrogallol. Change in the absorbance was recorded at 420 nm for 3 minutes after an initial lag of 30 seconds. The specific activity was calculated and expressed in units per mg of protein. One unit is defined as the amount of enzyme required to inhibit 50% autoxidation of pyrogallol.

### Determination of protein carbonylation (PC)

PC content was assayed by the procedure of Levine et al. [39]. Briefly, protein samples were incubated with 10 mM Dinitrophenylhydrazine (DNPH) in 2 M Hydrochloric acid (HCl) for 1 hour at room temperature, precipitated with 6% trichloroacetic acid (TCA) and washed three times by 6 M guanidine hydrochloride, 50% formic acid and centrifuged at 16,000g for 5 minutes to remove any trace of insoluble material and carbonyls were measured spectrophotometrically at 340 nm. The results were expressed as nanomoles of DNPH incorporated/mg protein, based on the molar extinction coefficient of 22,000 M^-1^cm^-1^.

### Assay of cathepsinL gene expression

Total RNA from the adult *Fasciola gigantica* control, curcumin and thymoquinone treated flukes was isolated by TRI^®^ reagent (Sigma Aldrich) and then reverse transcribed using reverse transcriptase enzyme (Fermentas) following the recommended protocols. The PCR reaction mixture of 25μl volume contained 2μg template cDNA, 1μM each of forward and reverse primers (Forward Primer: GATCGTTTGAGCCATGGAGT, Reverse Primer: CACATGTTTCCTCGGTTCCT) along with 1x PCR buffer and nuclease free water (Fermentas, Genetix, New Delhi, India) and the PCR reactions were carried out in Mastercycler nexus-gradient (Eppendorf). Each reaction was given an initial denaturation step of 15 min at 95°C followed by 35 cycles of denaturation at 94°C for 1 min, primer annealing at 58°C for 1 min, and primer extension at 72°C for 1 min. Each PCR reaction was terminated with a final extension step of 72°C for 15 min followed by cooling down to 4°C. The PCR products were analysed on a 1.5% agarose gel in1x TAE (40 mM Tris-acetate, 1 mM EDTA) buffer and compared their size with 100 bp dsDNA ladder (Thermo Scientific). The gel was photographed on a transilluminator (Genetix).

### Molecular modelling simulations

Basic methodology adopted for *in silico* molecular docking simulations was carried out according to Khan et al. [40]. Molecular docking simulations of CathepsinL from *Fasciola gigantica* interaction with curcumin and thymoquinone were performed with GOLDv5.1.1 program [41]. The crystal structure of CathepsinL (PDB Id: 2O6X) was downloaded from the Brookhaven Protein Data Bank. The two-dimentional (2D) structure of curcumin and thymoquinone were downloaded from Pubchem (pubchem.ncbi.nlm.nih.gov). 2D to three dimensional (3D) conversion was done with CORINAv2.6 (www.mol-net.de). The structure of CathepsinL was protonated in InsightII (www.accelrys.com). Genetic algorithm was implemented in GOLDv5.1.1 that was applied to calculate the possible conformations of the drug that binds to the protein. During the docking process, a maximum of 10 different conformations were considered for curcumin and thymoquinone. The conformer with the lowest binding free energy was used for further analysis. The binding energy of docked complexes was calculated using X-Score [42]. The amino acid residues making hydrogen bonding and hydrophobic interactions were calculated using Getneares, which is a tool available with DOCKv5.1.1 [43].

### Statistical analysis

The statistical analysis was carried out by using GraphPad prism 5 and the level of significance was calculated using one way ANOVA post hoc Tukeys test. All the experiments were carried out in triplicate and the results are presented as Mean ± S.E.M. and p<0.05 was considered significant.

## Results

### Worm motility and egg shedding

**Fig 1:**
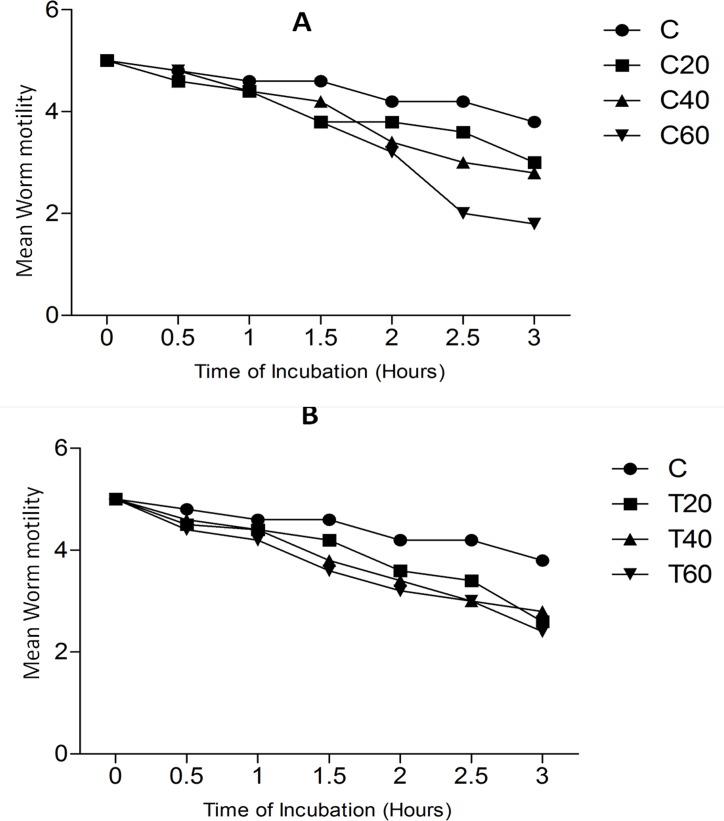
Mean worm motility of *Fasciola gigantica* treated with Curcumin (A) and Thymoquinone (B). Both compounds showing a time and concentration dependent decrease in worm motility and the highest concentration (60 μM) of both C and T results in significant inhibition (p < 0.05) in the motility of the worms as compared to the untreated controls.

The adult *Fasciola gigantica* worms treated with thymoquinone and curcumin appeared less motile with the increasing concentration of both the compounds, whereas, no major loss of activity in the control worms was observed. The decrease in the motility of worms treated with test compounds was both time and concentration dependent. The highest concentration (60 μM) of the two compounds produced a significant (p< 0.05) inhibition of the motility of worms as compared to the untreated controls Fig 1A and 1B. The analysis of data obtained on the *in vitro* egg shedding by the worms treated with the test compounds, thymoquinone and curcumin, showed an over all decreasing trend as compared to the untreated controls but this decline appeared to be insignificant and hence results are not shown.

### Surface topographical changes

**Fig 2:**
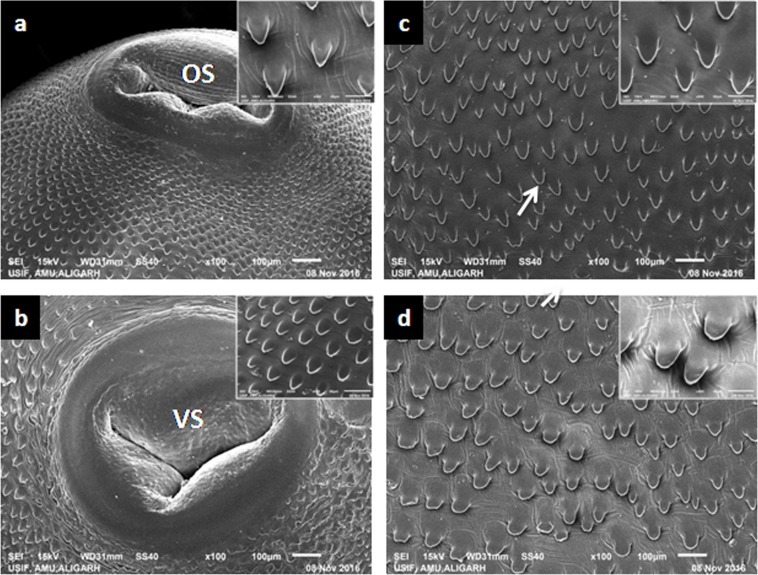
Scanning electron micrographs of adult *Fasciola gigantica* (Control) worms incubated in 0.2% DMSO for 3h at 37±1°C showing smooth oral sucker (OS), normal tegumental infoldings (a); intact and sharply pointed spines (a, inset); rounded and smooth ventral sucker (VS) surrounded by spines (b, inset); The posterior end of ventral surface with uniformly distributed tegumental spines (c); The dorsal surface showing tegumental infoldings interspersed with prominent spines (d, inset).

The tegumental topographical features of the control *F*. *gigantica* flukes incubated in dimethyl sulphoxide (DMSO) appeared normal as evident from the scanning electron micrographs showing smooth oral and ventral suckers, prominent spines and intact tegumental infoldings in the anterior region around the oral and ventral suckers. The posterior region on the ventral surface of the flukes exhibit evenly distributed spines and tegumental infoldings while distinct spines and tubercles were observed on the dorsal surface (Fig 2).

**Fig 3:**
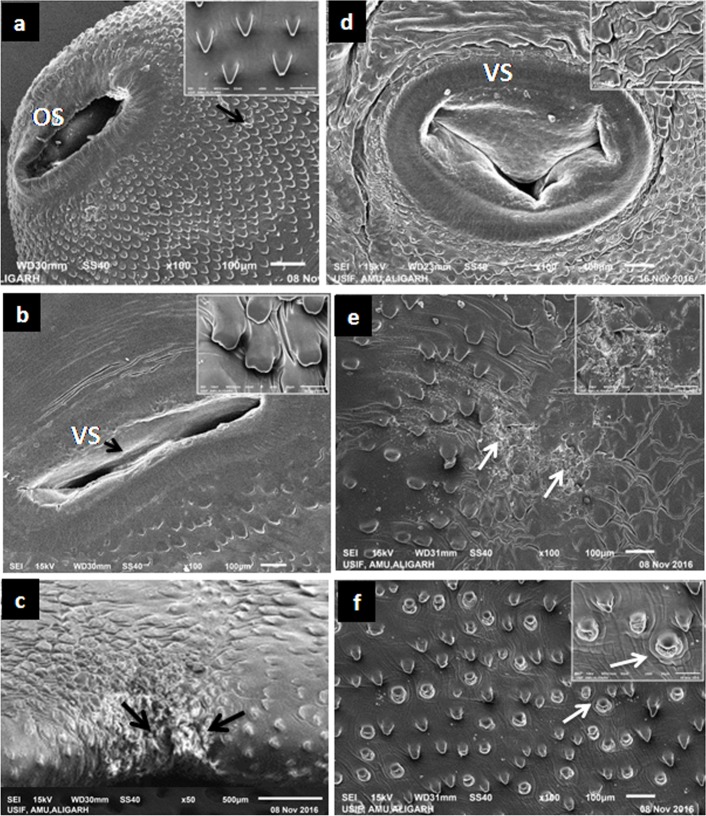
Scanning electron micrographs showing the effect of 40 μM (a, b, c) and 60 μM (d, e, f) curcumin on the tegumental surface of *F*. *gigantica*. The oral sucker (a) and the spines around it appeared normal, however, tegumental disruptions (b), swollen or bulging spines (b, inset) around disrupted acetabulum (VS), lesions around the excretory pore (c, arrows) and the erosions showing damaged posterior region (e) and dislodgement of huge number of spines from their sockets leaving pits (f, arrow).

**Fig 4:**
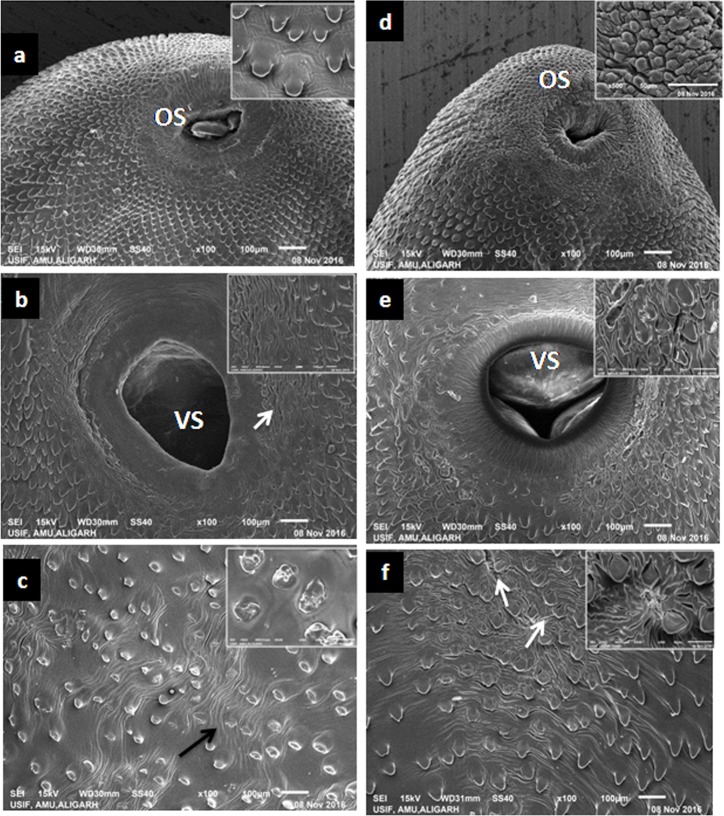
Scanning electron micrographs showing the effect of 40 μM (a, b, c) and 60 μM (d, e, f) thymoquinone on the tegumental surface of *F*. *gigantica*. Tegument appeared to be sunken around the oral sucker (a and d). Severely swollen spines and corrugated tegument were evident. Acetabulum appeared normal with wrinkled regions around them having deep furrows (b and e). The posterior regions showing severe dislodgement of spines (c, arrow) revealing the syncitium (f).

The tegumental area in and around the oral sucker region (Fig 3A) of the curcumin treated flukes did not show any significant change, while swelling, disruption of spines and tegumental infoldings were observed around the acetabulum in case of treated worms (Fig 3B and 3D). The tegumental damage was more prominent in case of worms treated with C-60 concentration as compared to the worms treated at C-40 (Fig 3C and 3E). The posterior end of the fluke showed severe blebbing as well as marked erosion of spines as evident in Fig 3E. The dorsal surface of the treated worms also appeared to be damaged and the disrupted spines could be clearly seen (Fig 3F). Similarly, the most pronounced tegumental changes were also observed following treatment of *F*. *gigantica* worms with 60 μM concentration (T-60) of thymoquinone as compared to the controls, however, 40 μM concentration of this compound produced less severe damage. The anterior regions of the thymoquinone treated worms (T-60) showed sunken tegument around the oral sucker (Fig 4A and 4D). The spines appeared sunken against the severely swollen and corrugated tegument. The region around the acetabulum appeared wrinkled, revealing deep furrows. A large number of spines appeared to be damaged, particularly at the anterior tips or eroded from the tegumental surface leaving pits at the site of attachments (Fig 4B and 4E). Similar tegumental changes were also observed in the posterior region of the treated worms (Fig 4C and 4F).

### Enzymatic analysis and cathepsinL expression

**Fig 5:**
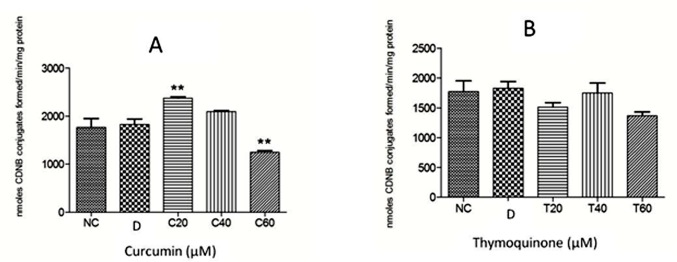
The glutathione-S-transferase (GST) levels in *Fasciola gigantica* worms treated with Curcumin (A) and Thymoquinone (B). The enzyme activity was initially stimulated but then declined significantly with the increasing concentration of curcumin, while the inhibitory effect of thymoquinone was statistically insignificant. NC: RPMI control, D: DMSO control; Curcumin at 20 μM (C20), 40 μM (C40) and 60 μM (C60) concentrations and thymoquinone at 20 μM (T20), 40 μM (T40) and 60 μM (T60) concentrations. All the experiments were carried out in triplicate ± S.E.M. **: p<0.01.

**Fig 6:**
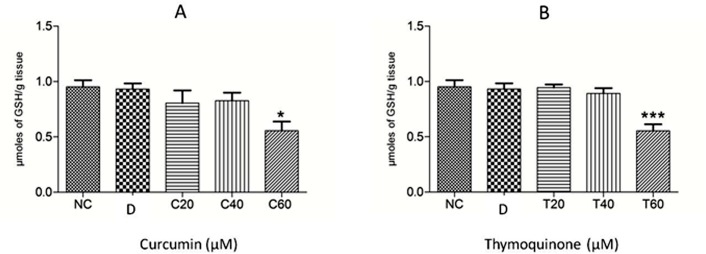
The reduced glutathione (GSH) levels in *Fasciola gigantica* worms treated with curcumin (A) and thymoquinone (B). The treatment of liver flukes with both curcumin and thymoquinone caused a significant decrease in the level of GSH that may be disrupting the redox-balance within the parasite. NC: RPMI control, D: DMSO control, Curcumin at 20 μM (C20), 40 μM (C40) and 60 μM (C60); and thymoquinone at 20 μM (T20), 40 μM (T40) and 60 μM (T60) concentrations. All the experiments were carried out in triplicate ±S.E.M. *: p<0.05, ***: p<0.001

**Fig 7:**
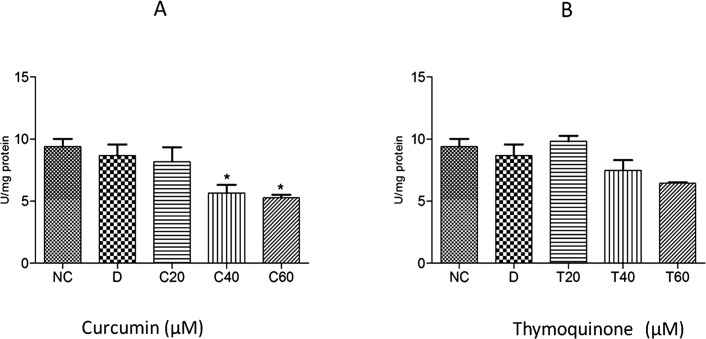
Superoxide dismutase (SOD) activity in the *Fasciola gigantica* adult worms treated with Curcumin (A) and Thymoquinone (B). Significant inhibition of SOD, particularly by curcumin will greatly impair the ability of worms to scavenge free radicals that will ultimately help in the elimination of parasites. NC: RPMI control, D: DMSO control, Curcumin at 20 μM (C20), 40 μM (C40) and 60 μM (C60) and Thymoquinone at 20 μM (T20), 40 μM (T40) and 60 μM (T60) concentrations. All the experiments were carried out in triplicate ±S.E.M., *: p<0.05

**Fig 8:**
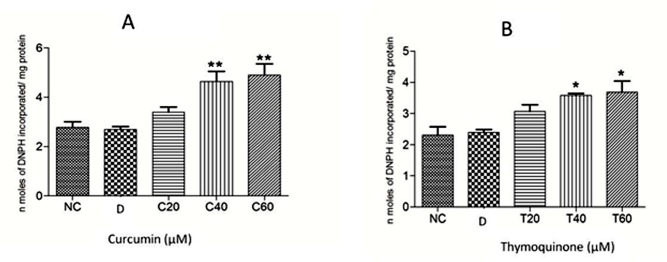
The level of protein carbonylation (PC) in *Fasciola gigantica* worms treated with different concentrations (20, 40 and 60 μM) of Curcumin (A) and Thymoquinone (B). Both the compounds produced significant increase in PC level in a concentration dependent manner. NC: RPMI control, D: DMSO control, Curcumin at 20 μM (C20), 40 μM (C40) and 60 μM (C60) concentration; Thymoquinone at 20 μM (T20), 40 μM (T40) and 60 μM (T60) concentration. Each experiment was repeated thrice + S.E.M. *: p<0.05, **: p<0.01

**Fig 9:**
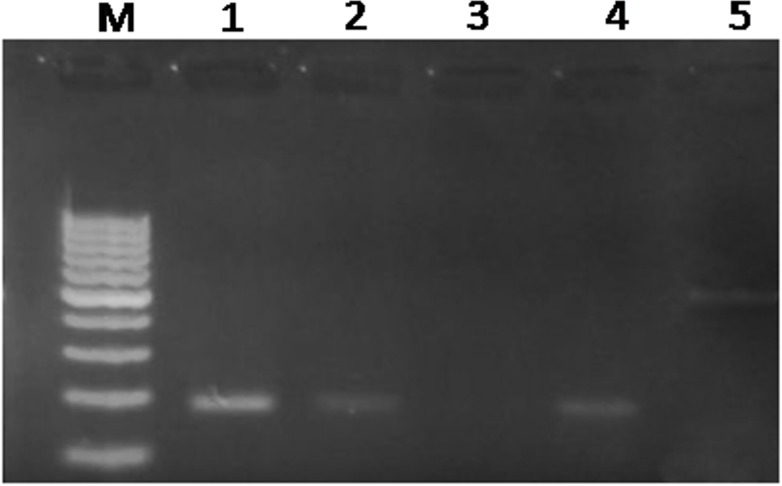
The effect of curcumin and thymoquinone on the expression of CatL gene of *Fasciola gigantica*. Thymoquinone at 40 μM significantly inhibited (lane 3) CatL gene expression in *F*. *gigantica*, while curcumin at this concentration appeared to be comparatively ineffective, reflecting a differential effect. M: DNA ladder (100bp), Lane 1: RPMI control, 2: DMSO control, 3: Thymoquinone treated sample, 4: Curcumin treated sample, 5: GAPDH was used as internal control.

The anthelmintic effect of curcumin and thymoquinone on *Fasciola gigantica* as evident from the inhibition of the key enzyme activities and *in silico* investigations in the present studies are summarized in Figs 5–9. It can be seen from the present results that curcumin initially stimulates the activity of the detoxifying enzyme, glutathione-S-transferase (GST), and then inhibits it significantly in a concentration dependent manner. Though thymoquinone also inhibited GST enzyme of the liver fluke but the effect appeared to be insignificant (Fig 5). However, the level of reduced glutathione was significantly inhibited by both curcumin as well as thymoquinone at the highest concentration (60 μM) used in this study (Fig 6). The activity of the anti-oxidant enzyme, superoxide dismutase (SOD) was also inhibited significantly by both the compounds understudy (Fig 7). The carbonylation, reflecting the oxidation of parasite proteins, was significantly increased in curcumin and thymoquinone treated worms with respect to increasing concentration of these compounds (Fig 8). Considering significant involvement of Cathepsins in the virulence of flukes, the effect of curcumin and thymoquinone on gene expression of CatL in *F*. *gigantica* was investigated. The results revealed a significant inhibition of CathepsinL gene expression by thymoquinone only indicating a differential effect of the two compounds understudy with respect to CatL gene expression in the tropical liver fluke, *F*. *gigantica* (Fig 9).

### *in silico* molecular interactions

**Fig 10:**
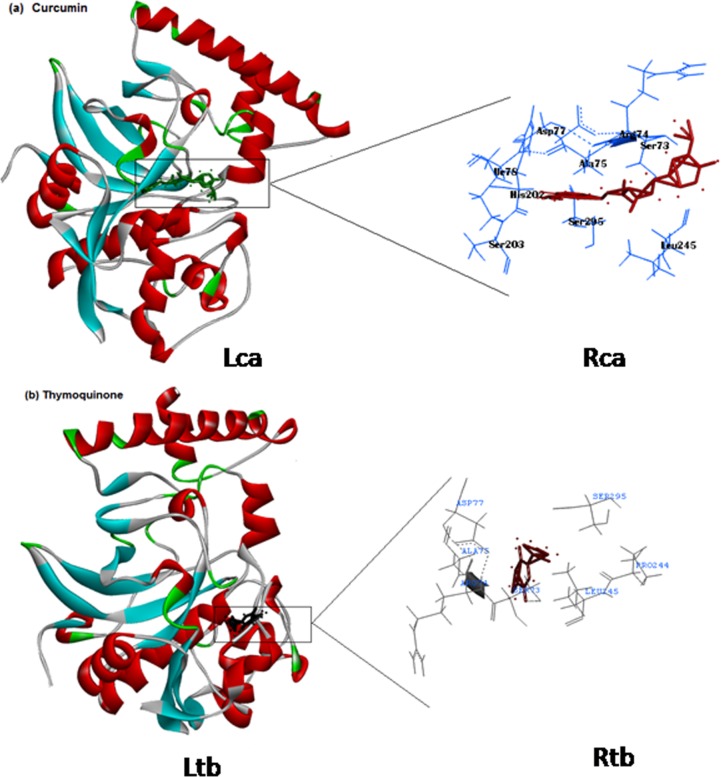
Computational model showing the binding interactions of Curcumin (a) (Lca, Rca) and Thymoquinone (b)(Ltb, Rtb) with CathepsinL of *Fasciola gigantica*. The hydrogen-bonding or electrostatic interaction acts as an "anchor" in the binding pocket (Lca, Ltb) and facilitate hydrophobic interaction of the chemical rings with the side chain of protein. Higher number of amino acids involved in the interaction placed curcumin (Rca) a little deeper in the binding site with CathepsinL compared to Thymoquinone (Rtb).

The results of *in silico* studies showed validation of the binding mode of curcumin and thymoquinone with CathepsinL of *F*. *gigantica* as per the amino acid residues predicted to be the part of the binding interactions in Fig 10, where Ile78, Ala75, Leu245, and Ala75, Leu245, Pro244 can make hydrophobic interactions with curcumin and thymoquinone, respectively. The interaction between CathepsinL and the tested molecules are not exclusively hydrophobic in nature since there are several ionic as well as polar residues (Asp77, Arg74, His202, Ser203, Ser295, Ser73 for curcumin and Asp77, Arg75, Ser295, Ser73, Pro244 for thymoquinone) in the proximity of the bound ligands, playing important role in stabilizing them via H-bonds and electrostatic interactions. Thus the hydrogen-bonding or electrostatic interaction acts as an “anchor”, intensely determining the 3D space position of the tested molecules in the binding pocket and facilitating the hydrophobic interaction of the chemical rings with the side chains of protein (Fig 10). Thus an overall comparison of the binding of curcumin and thymoquinone illustrates the stronger binding with the curcumin with cathepsinL. The higher number of amino acids involved in the interaction of curcumin with CathepsinL compared to thymoquinone is placing the curcumin little deeper in the binding site (Fig 10).

## Discussion

Helminthic infections pose a tremendous threat to the agri-based industries throughout the world. The global losses incurred due to fasciolosis alone are in the range of 3 billion USD [2]. In order to control this infection triclabendazole (TCBZ) remains the only drug of choice since it targets both the immature and adult stages of the *Fasciola* species. However, the emergence of resistance in liver flukes against the anthelmintics including triclabendazole [44–46] compromises the control strategies for these parasites [11]. The emerging TCBZ resistance in flukes from the human infections [47] is an additional challenge. Therefore, alternative measures including the use of plant derived natural products in search of novel drugs are being undertaken since plants are an important source for the discovery of anti-parasitic drugs because of the long association between the coexistence of parasites, humans and herbal remedies [48]. A wide variety of natural extracts and oils, purified compounds and herbal products have been used as anti-parasitic agents against the protozoan, monogenian, nematode, tapeworms and trematodes including liver flukes [23,25,27,28,32,49,50]. In the present study we have analysed the anthelmintic efficacy of curcumin and thymoquinone, which are derived from *Curcuma longa* and *Nigella sativa* respectively, against the tropical liver fluke *F*. *gigantica* under *in vitro* conditions. As evident from the present results both the compounds understudy exhibited chemopreventive and chemotherapeutic potential since they have caused significant inhibition of worm motility, severe disruption of the tegumental surface. The ultrastructural changes in the tegument of flukes have long been studied as an important parameter for the determination of efficacy of anthelmintic drugs and natural products [51–54]. The tegumental damage makes the parasite more vulnerable to the drug which can percolate within the worms, affecting several internal tissues and processes [55]. The tegumental disruptions including edema, severe blebbing and erosion of spines in the present study as revealed by scanning electron microscopy of *F*. *gigantica* worms following *in vitro* treatment with 40 μM and 60 μM each of curcumin and thymoquinone reflect the loss of tegumental transport and motility functions in the treated worms. It has been suggested that osmotic stress weakens the tegumental structures by affecting the β tubulin molecules [51] and the energy dependent Na^+^-K^+^ transport might be severely affected due to tegumental disruptions since internal Na^+^ and water level increases, leading to swelling of the syncitium as observed in nitroxynil treated *F*. *hepatica* worms [56,57]. The present results are in agreement with respect to tegumental disruptions with a previous study in which adult worms of *F*. *gigantica* were *in vitro* treated with the oil of *Nigella sativa* [32], however, the highest concentration of the two compounds under study produced maximum tegumental damage of the treated worms that also supports our study on antioxidant enzymes and protein carbonylation. The tegumental damage affects excretory/secretory processes along with trans-tegumental uptake, alters the signalling pathways and disturbs the metabolic processes [58]. Therefore, the thymoquinone and curcumin treated worms appeared to have lost their motility function within three hour of exposure of worms to the test compounds that may ultimately lead to paralysis and death of the worms, however this requires further studies to assess the effect of both the compounds for longer period of time.

Both thymoquinone and curcumin at the highest concentration significantly suppressed the activity of key free radical scavenging anti oxidant molecules like glutathione S transferase (GST), reduced glutathione (GSH), superoxide dismutase (SOD), altered protein carbonylation and inhibited the CathepsinL gene expression in adult flukes of *F*. *gigantica*. However, curcumin at low concentration (20 μM) caused a significant stimulation of GST activity in *F gigantica* but at higher concentrations enzyme activity was significantly inhibited contrary to a previous observation on *Setaria cervi* nematode where higher concentrations of curcumin enhanced GST and SOD activity, depleted GSH level and stimulated reactive oxygen species (ROS) generation leading to apoptosis [23]. Decreased GST and SOD activity is a direct effect of the compounds and not the destroyed parasites since worms were still alive but highly immotile at the end of the 3 hour incubation period. Parasitic infection causes increased production of reactive oxygen intermediates [59], resulting into an imbalance between pro- and anti-oxidant processes both *in vitro* and *in vivo* [60–62]. Helminths have a well developed antioxidant system which enables them to survive in the hostile environment generated by the host, where they neutralize the reactive oxygen species in order to establish a successful host-parasite relationship. Hence, an initial increase in GST activity at 20 μM concentration in the present study could possibly be a parasite protective response which could not be sustained by the worms at higher concentrations of curcumin, which is known to significantly elevate ROS level [23] that may lead to tegumental disruptions. However, thymoquinone did not inhibit GST activity significantly but its antiparasitic effect has been demonstrated in a previous study through immunomodulatory responses [28,63]. Targeting the key anti-oxidant molecules of helminths could render them vulnerable to the host generated reactive oxygen species and it will facilitate their elimination. GST and GSH functionally complement each other as GST mediates reaction of electrophilic metabolites of xenobiotics to the GSH (reduced glutathione) [64,65]. These enzymes function in a balanced way but as seen in the present results the level of GST and GSH decreased significantly showing failure of the counter mechanism in the parasite that would facilitate its elimination from the host.

The oil of *Nigella sativa* seeds, which contain thymoquinone as an active ingredient, caused surface tegumental damage in a number of helminth species including the tropical liver fluke *F*. *gigantica* [32], but the mode of action of thymoquinone is not known. In a previous study on cancerous cells thymoquinone has been reported to induce apoptosis where DNA damage occurred by possibly inhibiting the PAK-1/ERK-1/2 complex [66]. An increase in the level of free radicals can lead to DNA as well as protein damage [67,68] therefore, in the present study the oxidative damage of the parasite proteins as revealed by their carbonylation, which serve as the biomarker of protein damage, was significantly increased in *F*. *gigantica* worms following their treatment with both curcumin and thymoquinone in a concentration dependent manner. The maximum level of protein carbonylation was observed at 60 μM concentration of both curcumin and thymoquinone. The identification of carbonylated proteins could provide biomarkers for the oxidative damage inflicted by the test compounds in the parasites.

For the successful establishment within their host liver flukes secrete various enzymes (including Cathepsins) which are considered as the major component of the excretory secretory products and may be involved in immune evasion and establishment within the host beside other functions [13,69,70]. Cathepsins have been considered important vaccine targets as validated by gene silencing [14,18]. In the present study we have analysed the expression of cathepsinL gene in the tropical liver fluke, *F*. *gigantica* following *in vitro* treatment of worms with curcumin and thymoquinone. It was observed that thymoquinone significantly inhibited the CathepsinL gene expression (Fig 9), clearly indicating the potential of thymoquinone in the reduction of virulence of *Fasciola gigantica* that could occur by interfering with its invasive behaviour due to the inhibition of cysteine protease activity since CatL gene silencing experiments have revealed the loss of invasive potential of the worms [18]. While working with several cell lines Sethi et al. [71] have investigated the role of thymoquinone targeting the nuclear factor ĸ B activation pathway. It has been suggested that thymoquinone possibly inhibits the binding of NFĸB to DNA through modification of NFĸB proteins. Therefore it may be possible that similar phenomenon might be occurring in the liver flukes but it certainly requires further investigations. Despite the fact that DMSO is a safe drug solvent [72,73] it caused slight inhibition of CatL expression that was comparable to curcumin which did not affect CatL expression showing a differential effect of the two compounds with respect to CatL gene expression.

Further, molecular interactions of CathepsinL with thymoquinone and curcumin as validated through *in silico* study revealed the docking mode as per the amino acid residues predicted to be the part of the binding interactions (Fig 10) not only involve hydrophobic interactions with curcumin and thymoquinone but also several ionic as well as polar residues. Thus an over all comparison of the binding of curcumin and thymoquinone illustrates the stronger binding of curcumin with CatL. The higher number of amino acids involved in the interaction of curcumin with CatL compared to thymoquinone is placing the curcumin little deeper in the binding site. Hence, stronger interaction of the curcumin implicates its more pronounced effect on the enzyme activity. However, future studies are required to ascertain inhibition of CathepsinL activity of *F*. *gigantica* by thymoquinone to see that invasive behaviour and the virulent potential of the worms is significantly affected. Both the compounds, thymoquinone and curcumin are known to have synergistic effect [74] but nothing can be said at this stage about the combined effect of the two compounds since it has not been assessed in the present study.

## Conclusion

Taken together, it is concluded that both the compounds, curcumin and thymoquinone, used in the present study, inhibits the worm motility and severely disrupt the tegumental surface. These compounds also significantly suppress the detoxification and free radical scavenging ability of *F*. *gigantica* worms as well as their invasive ability by inhibiting the CathepsinL gene expression by thymoquinone and hence would affect their invasive activity. The molecular docking study further revealed strong interaction of these compounds with CathepsinL reflecting their efficacy. Therefore, both curcumin and thymoquinone appeared to be promising molecules for further investigations on their anthelmintic potential.
